# Reexamining microRNA Site Accessibility in *Drosophila*: A Population Genomics Study

**DOI:** 10.1371/journal.pone.0005681

**Published:** 2009-05-25

**Authors:** Kevin Chen, Jonas Maaskola, Mark L. Siegal, Nikolaus Rajewsky

**Affiliations:** 1 Center for Genomics and Systems Biology, Department of Biology, New York University, New York, New York, United States of America; 2 Max Delbrück Centrum für Molekulare Medizin, Berlin-Buch, Germany; Centre de Regulació Genòmica, Spain

## Abstract

Kertesz *et al.* (Nature Genetics 2008) described PITA, a miRNA target prediction algorithm based on hybridization energy and site accessibility. In this note, we used a population genomics approach to reexamine their data and found that the PITA algorithm had lower specificity than methods based on evolutionary conservation at comparable levels of sensitivity.

We also showed that deeply conserved miRNAs tend to have stronger hybridization energies to their targets than do other miRNAs. Although PITA had higher specificity in predicting targets than a naïve seed-match method, this signal was primarily due to the use of a single cutoff score for all miRNAs and to the observed correlation between conservation and hybridization energy. Overall, our results clarify the accuracy of different miRNA target prediction algorithms in *Drosophila* and the role of site accessibility in miRNA target prediction.

## Introduction

Population genomics has been suggested as a method of evaluating the accuracy of genome-wide predictions of *cis*-regulatory sites [1]–[4]. The idea is to use polymorphism data and population genetics techniques to estimate the level of purifying selection on predicted *cis*-regulatory sites genome-wide and to use this quantity as a proxy for the accuracy of the prediction algorithm. The underlying assumption is that an accurate prediction algorithm should identify functionally important sites that are likely to be under selective constraint. This is the same assumption underlying comparative genomics approaches but the population genomics approach is sensitive to natural selection of a different strength and on a different time scale. It is likely to become more useful in the future with the advent of high-throughput genome resequencing.

In this note we used a population genomics approach to reexamine the methods and data presented in Kertesz *et al.* [5]. There the authors presented a method for predicting miRNA binding sites in *Drosophila* using the score ddG = dG(duplex)–dG(open) where dG(duplex) is the hybridization energy of the miRNA to the binding site and dG(open) is the energy required to open the local RNA secondary structure around the binding site. The ddG score was used to rank all possible miRNA seed matches in 3′ UTRs (see [5] for details on the method). On a set of 190 experimentally validated target sites, the method was shown to perform more accurately than several leading methods, including Pictar [6], [7] and the method of Stark *et al.* [8], that do not use site accessibility but instead require conservation of seed matches between species. We found this result surprising because we expected that conservation would implicitly select for all sequence determinants of functional miRNA binding, including site accessibility. We therefore sought to corroborate the results of Kertesz *et al.* using a population genomics approach.

## Results

We used whole genome shot-gun sequence data from six inbred lines of *D. simulans* from the *Drosophila* Population Genomics Project [9] to estimate levels of polymorphism within *D. simulans* and divergence between *D. simulans* and *D. melanogaster* (Methods). To verify the accuracy of the data and our data processing methods, we first examined the patterns of polymorphism and divergence in miRNA genes (Table S1). These patterns have been established in previous studies of divergence across species [e.g. 10], [11] and within species [4], [12] and thus are a good test of data quality. We note that such an analysis was not possible in our previous study of SNPs in human miRNAs [1] or in miRNA resequencing studies in humans and Arabidopsis [13]–[15] because of the low rate of polymorphism in these species compared to *Drosophila*.

Our analysis of evolutionary patterns in miRNA genes confirmed the following hierarchy of selective constraint on the different parts of the miRNA precursor: seed>mature miRNA>star miRNA>loop>flanking control region (Text S1). Our analysis of indel patterns also confirmed that *D. simulans* miRNAs are more strongly depleted of indels than nucleotide substitutions compared to flanking control regions (Text S1), as previously observed between mammalian species [11]. A notable observation from our analysis is that the miRNA precursor loop length is under stabilizing selection since we observed a strong depletion of indels in the loop relative to flanking control regions (Table S2, Text S1) (one-sided Z test, insertions Z>3.4, P-value<0.0003, deletions Z>3.9, P-value<4.8e-5). This suggests that miRNA precursor loop length is functionally important, consistent with previous experimental [16] and computational [17] data.

**Figure 1:**
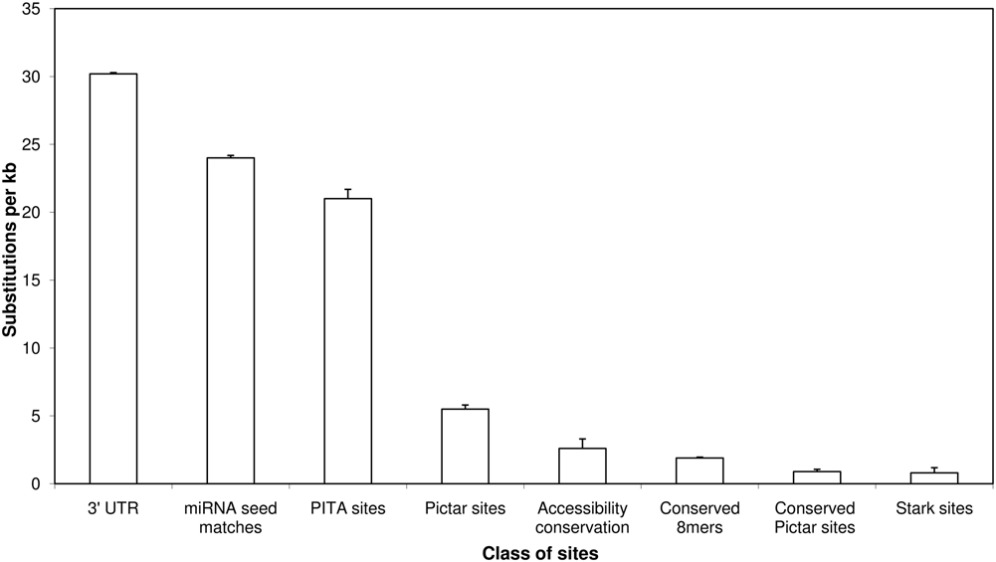
Substitution density in different classes of sites. From left to right: 3′ UTR, miRNA seed matches (sequences in 3′ UTRs reverse-complementary to bases 1–7 or 2–8 of mature miRNAs), PITA sites (top 15000 sites ranked by ddG, 0/0 set), Pictar sites (S1 set), Accessibility conservation (conserved but not necessarily aligned sites with ddG<−7 in each of 4 species), Conserved 8mers (conserved and aligned in 4 species), Conserved Pictar sites (S1 anchors, i.e. only conserved S1 sites), Stark sites (Branch Length Score = 0.9).

Having studied the evolutionary patterns of miRNA genes, we next reexamined the data presented in [5] that showed higher accuracy for PITA in comparison to the other miRNA target prediction methods using the area under the curve (AUC) metric applied to 190 previously validated miRNA targets. In contrast to those data, we found that Pictar [6.7] and the method of Stark *et al.* [8] (hereafter referred to as the “Stark method”) had significantly higher accuracy than PITA as quantified by three measures of selective constraint: SNP density (Z>19.6, P-value 0), substitution density (Z>26.6, P-value 0) and the McDonald-Kreitman test (P-value 5e-7) [18] (Methods, Figure 1, Table S3). Pictar and PITA had similar sensitivity, defined here as the total number of predicted targets (Table S3). We hypothesize that the discrepancy between our results and those of [5] is due to a systematic bias in the choice of the 190 validated targets in [5]: many of those targets may have been selected for experimental validation because they were predicted by computational methods based on conservation. Since we found that the three measures of selective constraint we used were entirely consistent across all the different data sets (Table S3) we report only P-values for substitution density in the rest of this note.

We validated the results of Figure 1 in three ways. First, to account for possible sequence-dependent mutation rate biases, we used published mutation rates of different bases in *Drosophila* [19] to correct the raw SNP and substitution densities (Methods). This correction decreased the difference between the selective constraint inferred on PITA-predicted targets versus Pictar and Stark-predicted targets by 7–8.5% but did affect our overall conclusions. Second, we explored the parameter space of the three algorithms by varying the number of species used by Pictar (4 vs. 6 species), the Branch Length Scores of the Stark method [8] and the accessibility settings of PITA (0/0 vs. 3/15 settings) (Methods). Our results showed that conservation outperformed site accessibility as estimated by PITA over a wide range of conservation and accessibility settings (Table S3). Since the two accessibility settings behaved very similarly in our analysis, we present only results for the 0/0 set for the rest of this note. Third, we compared our results to other studies that compared miRNA target prediction methods using protein abundance data following miRNA transfection or knock-down [20], [21]. These quantitative proteomics methods also showed higher accuracy for conservation-based approaches compared to other methods, including PITA.

Since PITA does not use conservation, we next tested if PITA outperformed a simple baseline method: a naïve seed-match procedure that predicts all sequences reverse-complementary to bases 1–7 or 2–8 from the 5′ end of a mature miRNA as a target site. We found that PITA indeed improved on the naïve method (Table S3, P-value<0.00003) but in the process we noticed that the set of PITA predicted targets was highly biased towards a small number of miRNAs. Such a bias can be caused by the use of a single cutoff score to rank all candidate miRNA sites in the genome, as opposed to fitting a separate cutoff score for each miRNA individually (e.g. RNAhybrid [22], Pictar [6], [7]). Neither approach is obviously superior because the appropriate cutoff for each miRNA ultimately depends on the cellular concentration of the miRNA averaged over various tissues and developmental stages, a quantity that is not currently available. Nonetheless, it is worth noting that when applied genome-wide, either method could potentially cause strong biases in the subset of miRNAs selected.

We hypothesized that deeply conserved miRNAs, defined as miRNAs conserved in vertebrates, *Drosophila* and nematodes [23], have stronger hybridization energy to their targets than other miRNAs, perhaps because they have been optimized towards stronger hybridization energy. Given their extreme conservation we expected that these miRNAs and their targets would be functionally important. Indeed we found many more binding sites for deeply conserved miRNAs among the top PITA targets ranked by dG(duplex) (39%) and ddG (33%) compared to dG(open) (20%) or local AU content (21%), a simple alternative method of estimating site accessibility (Methods). Deeply conserved miRNAs accounted for 29% of all seed matches. This pattern was also clear when we relaxed the criterion to miRNAs conserved in *Drosophila* and either vertebrates or nematodes (data not shown). We also found that ranking by dG(duplex) alone performed better than the naïve method (P-value 0.0002) while ranking by dG(open) alone performed slightly worse, though not significantly so (P-value 0.16) (Methods). We concluded that the primary determinant for PITA's better performance than the naïve method was hybridization energy, not accessibility, along with the observation that deeply conserved miRNAs have stronger hybridization energy to their targets than other miRNAs.

At a practical level, one could consider combining conservation, hybridization energy and site accessibility to predict miRNA targets. We thus tested two simple implementations of this idea. First, we simply ranked conserved Pictar sites by their PITA score. We observed an increase in the selective constraint among the highly ranked sites but were unable to make statistically significant statements due to the small amount of polymorphism data available (Table S3). Second, we predicted sites using conservation of accessibility (Methods). Briefly, we required a seed match in each species with a ddG below a threshold that we varied from 0 to −7 kcal/mol. However, to increase sensitivity and the amount of polymorphism available for analysis, we did not require the binding sites to be aligned but just that they appear anywhere in each orthologous 3′ UTR. We found a marginally significant trend for sites predicted using the −7 kcal/mol threshold to improve on sites predicted using the 0 kcal/mol threshold (P-value = 0.09, Methods). This result suggests that using conservation of accessibility gives a small improvement in predicting miRNA targets though we do not rule out that more sophisticated techniques could lead to a larger improvement.

## Discussion

In summary, our population genomics study produced three main findings: first, miRNA precursor loop length is under stabilizing selection in *D. simulans*; second, the relative accuracy of the miRNA prediction algorithms evaluated in Kertesz *et al.* [5] may require some revision; third, the hybridization energy of deeply conserved miRNAs to their targets tends to be stronger than that of other miRNAs.

Several methods of evaluating the accuracy of miRNA target prediction algorithms are commonly used. These include comparative genomics [24], [25], quantification of mRNA or protein abundance following miRNA over-expression or knock-down [19], [20], [26], immunoprecipitation of a RISC component (i.e. the protein complex that binds the miRNA) followed by analysis of the bound mRNAs by sequencing or microarrays (e.g. [27], [28]) and experimental validation of individual miRNA targets (e.g. using luciferase assays) [reviewed in 29]. The population genomics approach has several advantages over these methods. First, unlike comparative genomics, it is applicable to species-specific miRNA sites. Although it is difficult to estimate the number of species-specific sites under selection, previous work suggested that it is at least on the same order of magnitude as conserved sites [1]. Second, experimental approaches only test if the expression of a gene is repressed and not if the expression difference has a downstream effect on phenotype. Moreover, some experiments are performed under non-physiological conditions, e.g. over-expression of the miRNA or assay in a heterologous cell type. The population genetics approach examines the footprint of natural selection which implies a selectable phenotype, possibly even a subtle one that cannot be assayed in the lab. Third, unlike the experimental approaches that focus on a few miRNAs, experimental conditions or targets, it evaluates all miRNA binding sites in the genome.

Conversely, the population genomics approach suffers from its own disadvantages. One major disadvantage is that the amount of polymorphism in a population is typically small and therefore allows only an aggregate estimate of the accuracy of all miRNA binding sites. It is currently not possible to estimate the accuracy of targets for a particular miRNA, let alone a particular binding site. This situation could change given a quantum leap in sequencing technology that would allow a much larger number of genomes to be analyzed and thereby provide accurate estimates of low-frequency polymorphisms. A second disadvantage is that population genomics methods typically make implicit mathematical assumptions about the structure of populations (e.g. random mating) and genomes (e.g. uniform mutation rates) that may be inappropriate in some situations. In the context of the *D. simulans* data, one question is how well the six inbred lines are modeled as a randomly mating population. Overall the population genomics approach should be considered complementary to other approaches.

This study extends our previous analysis of genotyped human SNPs in miRNA binding sites [1] in several ways. First, the human SNP data are known to suffer from ascertainment bias (e.g. SNPs in non-synonymous sites were over-sampled) [30] and there was the possibility that some bias remained in spite of our controls. Second, SNP data are only a subset of all the polymorphisms across the genome. Third, the SNP data did not contain rearrangements such as indels. Fourth, we extended the analysis to an important model organism for population genetics, *D. simulans*. Fifth, we were able to study the evolution of miRNA genes whereas we did not have enough data to do this in humans. The current study confirmed the result from [1] that conserved miRNA sites are under strong negative selection even compared to other conserved 3′ UTR 8mers (P value<0.00007 in 4 species). However, unlike in humans [1], the naïve seed match method showed a signal of selective constraint relative to 3′ UTRs in *D. simulans* (P value<0.0006). Plausible reasons for this difference include the larger effective population size of *Drosophila*, longer 3′ UTRs in humans leading to more spurious seed matches and more non-conserved miRNAs annotated in humans than *Drosophila*.

While it was unsurprising that selective constraint on conserved miRNA sites was stronger than on non-conserved miRNA sites and selective constraint on accessible sites fell between these two extremes, the aim of our study was to determine the precise magnitude of the differences in selective constraint between these different data sets. In particular, we found significantly higher selective constraint on conserved miRNA sites than on accessible miRNA sites as computed by PITA.

## Materials and Methods

### Data

We used miRNA gene annotations from Rfam 10.0 [31] and supplemented them with annotations from [32]. We obtained *D. simulans* genome sequence data from the *Drosophila* Population Genetics Project (http://www.dpgp.org) [8]. We downloaded Pictar miRNA target predictions [33] from the UCSC genome browser [34]. We downloaded PITA miRNA target predictions and the PITA executable [5] from http://genie.weizmann.ac.il/pubs/mir07/index.html and miRNA target predictions from Stark *et al.* [7] from http://compbio.mit.edu/fly/motif-instances.

There are two sets of Pictar predictions: the S1 set uses conservation in *D. melanogaster*, *D. yakuba*, *D. ananassae and D. pseudoobscura* and the S3 set uses conservation in these four species as well as *D. mojavensis* and *D. virilis*. “Anchors” are conserved miRNA sites while the full S1 or S3 set also contains some species-specific miRNA sites. There are also two sets of PITA predictions: the 0/0 set does not require unpaired bases flanking the miRNA sites, while the 3/15 set requires 3 bases upstream and 15 bases downstream of the miRNA sites to be unpaired. These sets of parameters were learned from the training data in [5]. The Stark predictions have a BLS (Branch Length Score) parameter which refers to the fraction of total branch length on the phylogenetic tree on which the miRNA site is conserved.

We mapped all predicted miRNA target sites to genomic coordinates (*D. melanogaster* Release 4). Since a significant fraction of genes do not have experimentally supported 3′ UTRs, some algorithms simply use a fixed amount of genomic sequence downstream from the stop codon as the 3′ UTR. This procedure can lead to significant differences between different sets of miRNA target predictions so we considered only miRNA binding sites in annotated RefSeq 3′ UTRs. 3′ UTR alignments and RefSeq mRNA annotations were processed as previously described [6].

### Processing of D. simulans genome sequence data

Because of the low coverage of the shotgun sequence data and the variable number of lines sequenced across the genome, we chose not to estimate allele frequencies but only the presence or absence of SNPs and substitutions. Since low-coverage shotgun sequence data is prone to sequencing errors, we discarded bases with quality score <16, which corresponds to an error probability of ∼20%. We assume a base in *D. simulans* is the same as the *D. melanogaster* allele unless there is a different base passing the threshold score in at least one line. This assumption is correct in ∼95% of the cases, since the sequence divergence between the two species is ∼5% [8]. If there is a different *D. simulans* base, we assume that the base is a fixed substitution in *D. simulans* unless there is at least one other base passing the threshold score. This assumption is correct in ∼99% of cases since the polymorphism rate in *D. simulans* is ∼1% [8]. For insertions, we compared the minimum score of any base in the insertion to the threshold. Although the exact substitution and indel rates we report are sensitive to our choice of threshold, we based our conclusions only on the relative rates in different functional classes of nucleotides which are not biased by the choice of threshold since there is no reason to expect different functional classes to have different rates of sequencing error. Error bars in all tables and figures represent one standard deviation from a binomial distribution: square-root [np(1-p)] where n is the number of bases and p is the probability of the mutation falling into a particular segment. For Table S3 we made the additional approximation 1-p≈1.

### Population genomic tests

Lower SNP and substitution densities are consistent with stronger negative selection but these measures can be affected by variation in the mutation rate across the genome, for example due to base composition. One way to eliminate biases caused by mutation rate variation is to compare the ratio of fixed substitutions to polymorphisms using a Chi square test within the framework of the McDonald-Kreitman (MK) test [17] because mutation biases are expected to affect substitutions and polymorphisms equally. Although the MK test can be biased when used on a set of a genes with different genealogies, it is not biased when there is free recombination between all SNPs, an assumption we find reasonable for miRNA binding sites scattered across the genome and for *Drosophila*, in which the extent of linkage disequilibrium is generally low.

To compare the selective constraint on different classes of sites, we used two statistical tests. First, we used a one-sided Z test on the substitution or SNP density since for large sample sizes the distribution of the difference in substitution or SNP densities is approximately normally distributed. Selection is expected to affect divergence more strongly than polymorphism (an expectation realized in our data) so we mainly presented P-values for substitution density in the main text. Second, we used a Chi square test to compare the ratios of substitutions to polymorphisms of the two classes, applying the logic of the MK test.

### Comparison of miRNA target site predictions

For the comparison between Pictar, Stark and PITA, the specific parameters we compared were Pictar S1 anchors, Stark BLS score 0.9 and PITA 3/15 set. See the section *Data* above for details of these three sets. As shown in Table S3, we find entirely consistent results when varying the conservation parameters (i.e. SNP and substitution densities decreased with increasing cross-species conservation) and there was little difference between the different accessibility settings of PITA (3/15 vs. 0/0).

For the AU content analysis, in the main text we reported values for AU content in a window of 100-nt surrounding the binding site. We repeated our analysis for windows in the range 40–100-nt in increments of 20-nt and established that the ranges of the substitution density (28.8–30.2 per kb) and SNP densities (21.0–22.9 per kb) were small.

For the comparison of top PITA sites ranked by ddG, dG(duplex), dG(open) and AU content, we took the top 15000 sites as suggested by [5]. For all the target predictions, the measure of sensitivity used is the number of targets predicted and is not based on a reference set of validated targets.

### Correction for sequence dependent mutation bias

The top 15000 PITA sites (both 0/0 and 3/15 sets) have higher GC content than all miRNA seed matches (51–54% for PITA vs. 37% for all miRNA seed matches). Previous studies of the neutral mutation pattern in Drosophila using dead-on-arrival non-LTR retrotransposable elements in euchromatic regions suggested that the neutral mutation rate of G's and C's is 50% higher than A's and T's [18]. Although these mutation rates were not estimated separately for transcribed sequences, we found that the patterns in 3′ UTRs for SNPs and rooted substitutions (using *D. yakuba* as an outgroup) were comparable to the previous estimate (30% and 55% respectively). We thus used the 50% rate to correct the constraint estimates we computed for PITA sites, and we estimate that constraint on the top 15000 PITA sites is 7–8.5% higher than recorded in Table S3. Nonetheless, this correction does not affect the overall conclusions of our study.

### MiRNA site accessibility analysis

To predict miRNA sites using conservation of miRNA site accessibility, we used PITA with default parameters to predict miRNA sites individually in orthologous 3′ UTRs from *D. melanogaster*, *D. yakuba*, *D. ananassae* and *D. pseudoobscura*, the same four species used in the S1 settings of the Pictar miRNA site predictions. We considered a gene to be targeted by a miRNA if there was a miRNA site (not necessarily aligned) with ddG below a certain threshold in all four orthologous 3′ UTRs. We took thresholds of 0, −1, −3, −5 and −7 kcal/mol. We chose −7 kcal/mol because it is roughly the cutoff used for the top PITA 3/15 set of targets (−7.16 kcal/mol). For 3′ UTRs with more than one site for the miRNA, we took the minimum ddG.

## Supporting Information

Table S1 List of microRNAs removed from the analysis
(0.15 MB PDF)

Table S2 Insertions and deletions in microRNA genes
(0.26 MB PDF)

Table S3 Substitution density, SNP density and McDonald-Kreitman ratios of different classes of sites
(0.05 MB DOC)

Text S1 Evolutionary Patterns in MicroRNA Genes
(0.04 MB DOC)
