# Supplementary material for: Reexamining microRNA Site Accessibility in Drosophila: A Population Genomics Study
Source: PLoS One. 2009 May 25;4(5):e5681. doi: 10.1371/journal.pone.0005681 (PMC2682560; doi:10.1371/journal.pone.0005681)
Supplement: Table S1 — List of microRNAs removed from the analysis (0.15 MB PDF) [file pone.0005681.s001.pdf]

dme-mir-303	Rejected
dme-mir-979	More than 0.5 non-conserved;<br>52 deletions in md106 and sim4+6
dme-mir-982	16 substitutions in w501+f, only 3 consecutive pairs
dme-mir-983-1	15 substitutions in w501+f sim4+6 and md199, only 1 consecutive pair;<br>47 deleted, almost all in w501+f, some in sim4+6 (and md199)
dme-mir-983-2	More than 0.5 non-conserved;<br>53 deleted, all in w501+f
dme-mir-984	More than 0.5 non-conserved;<br>78 deleted, all in w501+f (no-other information present?)
dme-mir-985	30 deletions in sim4+6 in two blocks: 20, 10nt
dme-mir-997	21 substitutions, 3 consecutive pairs, lots of small insertions in various lines
dme-mir-1007	not sequenced
dme-mir-929	not sequenced
dme-mir-954	not sequenced (chrIV)
dme-mir-957	not sequenced
dme-mir-958	not sequenced
dme-mir-975	not sequenced
dme-mir-976	not sequenced
dme-mir-977	Partially sequenced
dme-mir-991	not sequenced
dme-mir-992	Partially sequenced;<br>More than 0.5 non-conserved
dme-mir-iab4as	not sequenced

Supplementary table 1: List of discarded miRNA
