# Supplementary material for: Reexamining microRNA Site Accessibility in Drosophila: A Population Genomics Study
Source: PLoS One. 2009 May 25;4(5):e5681. doi: 10.1371/journal.pone.0005681 (PMC2682560; doi:10.1371/journal.pone.0005681)
Supplement: Table S2 — Insertions and deletions in microRNA genes (0.26 MB PDF) [file pone.0005681.s002.pdf]

## Insertions

In the 5th column, numbers in brackets are quality scores.

| | | | | |
|---|---|---|---|---|
| mir-280 | 3809949 | md106=>"a" | Mature | Dubious: run of As (32) |
| | 3809934 | md106=>"t" | Mature | Dubious: run of Ts (19) |
| mir-313 | 16099094 | md199=>"t" | Loop | Dubious: run of Ts (57) |
| mir-2a-2 | 19565434 | md106=>"t" | Mature | Dubious: run of Ts (16) |
| mir-286 | 15176346 | sim4+6"=>"c" | Loop | |
| | | c1674=>"c" | Loop | |
| | | w501+f"=>"c" | Loop | |
| | | md199=>"c" | Loop | |
| | | md106=>"c" | Loop | G insertion; quality ok (90) |
| mir-1003 | 20484338 | newc=>"g" | Star | |
| mir-1010 | 18118635 | md199=>"att" | Loop | |
| | | sim4+6=>"att" | Loop | |
| | | md106=>"att" | Loop | |
| | | w501+f"=>"att" | Loop | |
| mir-990 | 12410154 | md199=>c | Mature | |
| mir-989 | 9661342 | md106=>c | Loop | |
| mir-964 | 5642103 | c1674=>tgtggttatgataaatca | Flank | |
| | 5642103 | md199=>cgtggttatgataagtca | Flank | |
| | 5642103 | sim4+6=>tgtggtt | Flank | |
| | | w501=>tgtggtt | Flank | |
| | 5642105 | c1674=>c | Flank | |
| | | md199=>c | Flank | |
| | 5642109 | sim4+6=>agtcaatcaaaa | Flank | |
| | | w501+f=>agtcaatcaaaa | Flank | |

| | | | | |
|---|---|---|---|---|
| mir-966 | 6045680 | w501+f=>aatgtcg | Mature | |
| mir-967 | 12460020 | sim4+6=>ct | Star | |
| | 12460009 | w501+f=>actctc | Flank | |

## Deletions

| | | | | |
|---|---|---|---|---|
| mir-303 | 4211476 | sim4+6, w501+f, c1674 | Mature 4 | |
| mir-276b | 10293784 .. 10293785 | md106, sim4+6, newc, w501+f, md199, c1674 | Loop | fixed? |
| | 10293735 | md106, sim4+6, newc, w501+f, md199, c1674 | Flank | fixed? |
| mir-5 | 15176057 | md106, sim4+6, w501+f, md199, c1674 | Loop | polymorphic |
| mir-9b | 16694722 .. 16694723 | c1674 | Loop 3' | polymorphic |
| | 16694714 .. 16694715 | md199 | Loop 5' | polymorphic |
| mir-133 | 20604269 | md106 | Mature 20 | polymorphic |
| mir-288 | 20605992 | md106 | Loop / end mature | polymorphic |
| mir-1012 | 22687100 | sim4+6, md199, w501+f, c1674 | Loop | polymorphic |
| mir-1005 | 4343724 | md106, sim4+6, w501+f, c1674 | Loop | polymorphic |
| mir-986 | 3956805 | newc | Lower | polymorphic |
| mir-961 | 5641273 | c1674, md106, sim4+6, w501+f | Lower | fixed? |
| mir-972 | 19385103 | c1674, md106, md199, sim4+6, w501+f | Lower | fixed? |
| mir-973 | 19385466 .. 19385468 | md106, md199, sim4+6, w501+f | Lower | fixed? |

| | | | | |
|---|---|---|---|---|
| | 19385525 | md106, md199, sim4+6, w501+f | Loop | fixed? |
