# Supplementary material for: Reexamining microRNA Site Accessibility in Drosophila: A Population Genomics Study
Source: PLoS One. 2009 May 25;4(5):e5681. doi: 10.1371/journal.pone.0005681 (PMC2682560; doi:10.1371/journal.pone.0005681)
Supplement: Table S3 — Substitution density, SNP density and McDonald-Kreitman ratios of different classes of sites (0.05 MB DOC) [file pone.0005681.s003.doc]

Supplementary Table 3. Polymorphism and divergence (only single nucleotide substitutions) in predicted miRNA binding sites from PicTar , PITA and Stark *et al.* . See Methods for details of the different sets of target predictions.

| Functional class | Bases | Substitutions/kb | SNPs / kb | Subs / Snps |
| --- | --- | --- | --- | --- |
| 3’ UTRs | 3971859 | 30.2 ± 0.09 | 18.9 ± 0.07 | 1.60 |
| Conserved 8mers  (4 species) | 480077 | 1.9 ± 0.06 | 3.7 ± 0.08 | 0.51 |
| Conserved 8mers  (6 species) | 179437 | 1.0 ± 0.07 | 2.3 ± 0.11 | 0.43 |
| Conserved 7mers  (4 species) | 595343 | 4.8 ± 0.09 | 4.6 ± 0.09 | 1.04 |
| Conserved 7mers  (6 species) | 225968 | 3.0 ± 0.12 | 3.0 ± 0.15 | 1.00 |
| All miRNA seed matches | 704678 | 24.0 ± 0.19 | 14.9 ± 0.15 | 1.61 |
| PITA top 3/15 | 48780 | 21.8 ± 0.67 | 14.9 ± 0.55 | 1.46 |
| PITA top 0/0 | 44417 | 21.0 ± 0.69 | 13.8 ± 0.56 | 1.52 |
| PITA top 0/0 conservation > 0.9 | 12764 | 5.7 ­± 0.67 | 5.0 ± 0.63 | 1.14 |
| PITA top 0/0 conservation <= 0.9 | 31700 | 27.1 ± 0.92 | 17.4 ­± 0.74 | 1.56 |
| PITA top 0/0, no 6mer seeds | 35649 | 21.3 ± 0.77 | 13.8 ± 0.62 | 1.54 |
| PITA top 0/0, no 6mer or G/U seeds | 11278 | 18.2 ± 1.27 | 12.3 ± 1.0 | 1.48 |
| Pictar S1 | 59881 | 5.5 ± 0.3 | 4.6 ± 0.28 | 1.20 |
| Pictar S1 anchors | 36012 | 0.9 ± 0.16 | 1.8 ± 0.22 | 0.50 |
| Pictar S3 | 26231 | 3.6 ± 0.37 | 3.9 ± 0.39 | 0.92 |
| Pictar S3 anchors | 20456 | 0.6 ± 0.17 | 1.4 ± 0.26 | 0.43 |
| Pictar S1 intersect with PITA top 0/0 | 5938 | 2.1 ± 0.59 | 3.5 ± 0.77 | 0.60 |
| Pictar S1 intersect with PITA top 3/15 | 6182 | 3.2 ± 0.72 | 3.0 ± 0.70 | 1.07 |
| Pictar S1 intersect with PITA top 0/0 taking all sites below a cutoff of -6 ddG | 25176 | 3.5 ± 0.37 | 3.1 ± 0.35 | 1.13 |
| Stark (BLS > 0.0) | 86039 | 23.2 ± 0.52 | 15.5 ± 0.42 | 1.50 |
| Stark (BLS > 0.2) | 38649 | 7.7 ± 0.45 | 7.0 ± 0.43 | 1.10 |
| Stark (BLS > 0.4) | 26493 | 6.2 ± 0.48 | 4.9 ± 0.43 | 1.27 |
| Stark (BLS > 0.6) | 19280 | 3.3 ± 0.41 | 3.2 ± 0.41 | 1.03 |
| Stark (BLS > 0.8) | 11086 | 1.8 ± 0.4 | 1.5 ± 0.37 | 1.20 |
| Stark (BLS > 0.9) | 5639 | 0.8 ± 0.38 | 1.2 ± 0.46 | 0.67 |
| Conserved sites (not aligned) (no ddG cutoff) | 30487 | 3.8 ± 0.35 | 3.8 ± 0.35 | 1 |
| Conserved sites (not aligned) (ddG < –1) | 22785 | 3.8 ± 0.41 | 3.7 ± 0.40 | 1.03 |
| Conserved sites (not aligned) (ddG < –3) | 16279 | 3.4 ± 0.46 | 3.6 ± 0.47 | 0.94 |
| Conserved sites (not aligned) (ddG < –5) | 10271 | 3.0 ± 0.54 | 3.6 ­± 0.59 | 0.83 |
| Conserved sites (not aligned) (ddG < –7) | 5331 | 2.6 ± 0.70 | 3.6 ± 0.82 | 0.72 |
