# Supplementary material for: Reexamining microRNA Site Accessibility in Drosophila: A Population Genomics Study
Source: PLoS One. 2009 May 25;4(5):e5681. doi: 10.1371/journal.pone.0005681 (PMC2682560; doi:10.1371/journal.pone.0005681)
Supplement: Text S1 — Evolutionary Patterns in MicroRNA Genes (0.04 MB DOC) [file pone.0005681.s004.doc]

Supplementary Note

## *Evolution of miRNA genes in D. simulans*

We mapped all *D. melanogaster* miRNA precursors from Rfam 10.1 to the six *D. simulans* lines using the *D. simulans* syntenic assembly produced by the *Drosophila* Population Genomics Project ([http://www.dpgp.org](http://www.dpgp.org/)). 141 out of 152 miRNAs had complete sequence coverage in the *D. simulans* assembly, while the other 11 miRNAs did not have complete coverage, typically because they are in heterochromatic regions. Of the 141 fully sequenced miRNAs, we identified eight miRNAs that had an unusually high number of differences between *D. melanogaster* and *D. simulans* (miR-303, miR-982, miR-983-1, miR-983-2 and miR-984, which form a cluster, as well as miR-979, miR-985, and miR-997). The high rate of divergence and the positions of the differences in the miRNAs strongly suggest that these miRNAs are not functional in *D. simulans* (see Supplementary Table 1 for details). Although we cannot completely exclude the possibility that these are functional miRNAs that have diverged in function in *D. simulans*, we chose to exclude them from the remaining analysis, leaving a set of 133 confidently identified *D. simulans* miRNAs. Regardless of whether the eight miRNAs are non-functional or highly diverged in *D. simulans*, these data demonstrate that miRNAs can evolve rapidly over short evolutionary distances.

The McDonald-Kreitman (MK) test is commonly used to infer directional selection by comparing the ratio of divergence to polymorphism in a putatively selected region (typically non-synoymous sites) to that in a putatively neutral region (typically synonymous sites). Adaptive mutations contribute more to divergence than to polymorphism, so an excess of divergence relative to polymorphism is indicative of positive selection. Conversely, weakly deleterious mutations contribute more to polymorphism than to divergence and so an excess of polymorphism is consistent with weak negative selection. We observed a high ratio of divergence to polymorphism in miRNA genes compared to control flanking regions (see next section, Chi square test, P value < 5e-5). This pattern is consistent with, but does not necessarily prove, the notion that at least some miRNAs, like protein coding genes, have evolved adaptively in *Drosophila*.

Despite the significant result from the MK test, caution in interpretation is warranted when applying the MK test on a set of genes with potentially different genealogies scattered across the genome because it is possible for the combination of a set of neutral MK tables to result in an MK table that rejects neutrality (see [1] for a detailed discussion). Indeed, we observed that one family in particular, miR-310-313, had an usually high amount of divergence and low level of polymorphism, both in the miRNA and control flanking regions. We noticed this family because there are two fixed substitutions each in the mature miRNA sequences of miR-312 and miR-313 (though not in the seed). Using the *D. yakuba* sequence as an outgroup, we found that substitutions in this region frequently occurred on both the *D. simulans* and *D. melanogaster* lineages and we verified that the pattern of polymorphism and divergence observed was not due to unusually low sequence coverage in this region.

Although the low level of polymorphism in this family is consistent with a selective sweep, another possible explanation for the pattern could be an unusually low recombination rate in the miR-310-313 region. Since accurate genome-wide recombination rates are not available for *D. simulans*, we used an estimate of the recombination rate in *D. melanogaster* . There is a significant correlation between *D. simulans* polymorphism and *D. melanogaster* recombination rates, suggesting that recombination rates between these two species are comparable. The recombination rate in this region was estimated to be 3.7 cM/Mbp, which is close to the highest rate on chromosome 2R (range: 0 to 3.76 cM/Mbp, average: 2.43 cM/Mbp) ‑­

(http://cgi.stanford.edu/~lipatov/recombination/recombination-rates.txt), thus we can exclude the effect of suppressed recombination at this locus. In summary, the pattern of divergence and polymorphism in this gene family is suggestive of one or more selective sweeps on the region containing the miR-310-313 family, though experimental tests of the functional changes are needed to exclude other possible non-selective explanations, such as demography. Taken together, our data indicate that miRNA genes have evolved rapidly between *D. melanogaster* and *D. simulans* and that in at least one case, evolution may have been adaptive. Our results are consistent with two recent studies on the evolution of miRNAs, including the miR-310-313 family, between *D. melanogaster* and *D. simulans* [2,3].

## *Variation in miRNA genes strongly correlates with miRNA precursor structure*

We used RNAfold [4] to predict the minimum free energy secondary structures of the miRNA precursors. Based on these predicted secondary structures and the annotated mature miRNA sequences from Rfam 10.1, we defined the following five segments of the miRNA gene: annotated mature region; the star-region that base-pairs to it, taking into account the stereotypical 2-nt 3'-overhang; the loop region consisting of the bases between the mature and star regions; the lower, base-paired segment; and the flanking region defined as the single-stranded flanks of the miRNA precursor (Supplementary Figure 1). In the case of mir-1017, this procedure produced a lower stem only downstream of the hairpin, so this segmentation was manually corrected, leading to a reduction by 10 counts of substitutions in the lower stem region.

MiRNA precursors can be divided into 5 regions: mature miRNA, miRNA star, loop, lower stem and flank (Supplementary Figure 1). Each of these regions is expected to experience different levels of selective constraint. We segmented each miRNA precursor into these five different regions using the predicted minimum free energy (MFE) secondary structure of the miRNA (Methods). We then tabulated all fixed and polymorphic substitutions and indels in the precursor miRNAs (Supplementary Tables 1 and 2, Supplementary Figures 1 and 2). We used 50-nt of flanking sequence on either side of the miRNA precursors as (putatively) neutral controls. Using control sequences in the local neighborhood of the miRNAs is important to control for variation in rates of polymorphism and divergence across the genome. There are no known functional constraints on the flanking sequences of miRNA precursors and since we observed increased selective constraint on miRNA genes compared to flanking sequences, the presence of functional elements in the flanking sequences would only make our approach more conservative. We did not include control sequences for the mirtrons (i.e. miRNAs that constitute an entire intron) since this would be exonic sequences which are unlikely to evolve neutrally.

We found the seed region (positions 2-8) of the mature miRNA to be under very strong selective constraint since we did not find any substitutions in this region. However, we found fixed substitutions in the first position of miR-960 and miR-973, consistent with the target model that the first position of the mature miRNA is not bound to the target mRNA . We also observed polymorphisms in the seeds of miR-133, miR-280, miR-966 and miR-990 that could be of functional interest since they are expected to affect miRNA targeting. Of these polymorphisms, we attempted to validate the one in miR-133 because it is the best studied of these four miRNAs. However, upon resequencing of the relevant *D. simulans* line, this was found to be a sequencing error (data not shown).

In addition to the seed, we detected strong selective constraint on the remainder of the mature sequence, with SNP density only ~5% relative to the controls (Supplementary Table 1). This level of constraint may indicate that either 3’ compensatory miRNA binding sites or regulatory elements in this region of the miRNA that mediate post-transcriptional control are more common than currently believed. Also, consistent with previous studies across species, selective constraint on the loop and miRNA flanking sequences was low compared to the rest of the miRNA, but still below the level of the controls (Supplementary Table 1).

Finally, we observed significant selective constraint on the star sequence, with SNP density ~13% that of controls. This pattern likely reflects base pairing constraints both to pair to the mature miRNA and to prevent the inappropriate strand from being incorporated into the RISC complex. An additional source of constraint is that the star sequence can be incorporated into the miRNA-mediated silencing complex and the scope of targeting by the star sequence is of interest. To address this question, we used the lower stem of the miRNA to model the selective constraint expected due to base pairing constraints and tested for excess constraint on the star mIRNA relative to the lower stem. We did not detect any excess constraint on the star miRNA either with respect to SNP and substitution density or with respect to the ratio of divergence to polymorphism (see below, Chi square test, P-value > 0.63).

## *Strong selection against insertions and deletions in miRNA genes*

An advantage of whole-genome shotgun sequence data over SNP data is the ability to study genome rearrangements, such as indel variation. Overall, we observe that indels are depleted in miRNA genes relative to control sequences, making indel depletion a good feature for identifying miRNAs (Supplementary Table 2). A similar pattern was previously observed using cross-species data in mammals [5].

It is an interesting question whether the size of the loop region of the miRNA is under selective constraint. It has been argued that the loop cannot be too big [6] otherwise it would appear to be single-stranded RNA and cause inappropriate *Drosha* processing while experiments have shown that small loops have impaired processing [7]. We observed a strong depletion of indels in the loop (Supplementary Table 2) (one-sided Z test, insertions Z > 3.4, P-value < 0.0003, deletions Z > 3.9, P-value < 4.8e-5). This pattern suggests the action of stabilizing selection on loop length, which in turn implies that the length of the loop is functionally important. We verified that this result was not due to a significantly elevated level of insertions in the flanking regions of miRNAs by checking that the rate of insertions in intergenic regions (8.9 / kb) was higher than in miRNA control regions (7.3 / kb). The rate of insertions across the entire genome was lower (6 / kb), presumably due to the lower rate of insertions in genes.

1. Shapiro J, Huang W, Zhang C, Hubisz M, Lu J, et al. (2007). [Adaptive genic evolution in the Drosophila genomes.](http://www.ncbi.nlm.nih.gov/pubmed/17284599?ordinalpos=2&itool=EntrezSystem2.PEntrez.Pubmed.Pubmed_ResultsPanel.Pubmed_DefaultReportPanel.Pubmed_RVDocSum) Proc Natl Acad Sci 104:2271-6.

2. Lu J, Shen Y, Wu Q, Kumar S, He B, et al. (2008) The birth and death of microRNA genes in Drosophila. Nat Genet 40: 351-355.

3. Lu J, Fu Y, Kumar S, Shen Y, Zeng K, et al. (2008) Adaptive evolution of newly-emerged microRNA genes in Drosophila. Mol Biol Evol 25: 929-938.

4. Hofacker I, Fontana W, Stadler P, Bonhoeffer S, Tacker M, et al. (1994) Fast Folding and Comparison of RNA Secondary Structures. Monatshefte f Chemie 125: 167-

188.

5. Lunter G, Ponting C, Hein J (2006) Genome-wide identification of human functional DNA using a neutral indel model. PLoS Comput Biol 2: e5.

6. Ruby J, Stark A, Johnston W, Kellis M, Bartel D, et al. (2007) Evolution, biogenesis, expression, and target predictions of a substantially expanded set of Drosophila

microRNAs. Genome Res 17: 1850-1864.

7. Han J, Lee Y, Yeom K, Nam J, Heo I, et al. (2006) Molecular basis for the recognition of primary microRNAs by the Drosha-DGCR8 complex. Cell 125: 887-901.
